# Characterization of the 4,6-α-glucanotransferase GTFB enzyme of *Lactobacillus reuteri* 121 isolated from inclusion bodies

**DOI:** 10.1186/s12896-015-0163-7

**Published:** 2015-06-09

**Authors:** Yuxiang Bai, Rachel Maria van der Kaaij, Albert Jan Jacob Woortman, Zhengyu Jin, Lubbert Dijkhuizen

**Affiliations:** Microbial Physiology, Groningen Biomolecular Sciences and Biotechnology Institute (GBB), University of Groningen, Nijenborgh 7, 9747 AG Groningen, The Netherlands; Department of Polymer Chemistry, Zernike Institute for Advanced Materials, University of Groningen, Nijenborgh 4, 9747 AG Groningen, The Netherlands; The State Key Laboratory of Food Science and Technology, School of Food Science and Technology, Jiangnan University, Wuxi, 214122 China

**Keywords:** *Lactobacillus reuteri*, 4,6-α-glucanotransferase, GTFB enzyme, Modified starch, Dietary fiber, Inclusion bodies

## Abstract

**Background:**

The GTFB enzyme of the probiotic bacterium *Lactobacillus reuteri* 121 is a 4,6-α-glucanotransferase of glycoside hydrolase family 70 (GH70; http://www.cazy.org). Contrary to the glucansucrases in GH70, GTFB is unable to use sucrose as substrate, but instead converts malto-oligosaccharides and starch into isomalto-/malto- polymers that may find application as prebiotics and dietary fibers. The GTFB enzyme expresses well in *Escherichia coli* BL21 Star (DE3), but mostly accumulates in inclusion bodies (IBs) which generally contain wrongly folded protein and inactive enzyme.

**Methods:**

Denaturation followed by refolding, as well as ncIB preparation were used for isolation of active GTFB protein from inclusion bodies. Soluble, refolded and ncIB GTFB were compared using activity assays, secondary structure analysis by FT-IR, and product analyses by NMR, HPAEC and SEC.

**Results:**

Expression of GTFB in *E. coli* yielded > 100 mg/l relatively pure and active but mostly insoluble GTFB protein in IBs, regardless of the expression conditions used. Following denaturing, refolding of GTFB protein was most efficient in double distilled H_2_O. Also, GTFB ncIBs were active, with approx. 10 % of hydrolysis activity compared to the soluble protein. When expressed as units of activity obtained per liter E. coli culture, the total amount of ncIB GTFB expressed possessed around 180 % hydrolysis activity and 100 % transferase activity compared to the amount of soluble GTFB enzyme obtained from one liter culture. The product profiles obtained for the three GTFB enzyme preparations were similar when analyzed by HPAEC and NMR. SEC investigation also showed that these 3 enzyme preparations yielded products with similar size distributions. FT-IR analysis revealed extended β-sheet formation in ncIB GTFB providing an explanation at the molecular level for reduced GTFB activity in ncIBs. The thermostability of ncIB GTFB was relatively high compared to the soluble and refolded GTFB.

**Conclusion:**

In view of their relatively high yield, activity and high thermostability, both refolded and ncIB GTFB derived from IBs in *E. coli* may find industrial application in the synthesis of modified starches.

## Background

GTFB is a novel family GH70 enzyme isolated from the probiotic bacterium *Lactobacillus reuteri* 121 [1]. The GTFB protein sequence is similar to that of the glucansucrase GTFA of *L. reuteri* 121 (61 % similarity), but these structurally related proteins have different enzymatic activities [2]. Unlike the common GH70 glucansucrases, GTFB is inactive on sucrose, but acts as a 4,6-α-glucanotransferase: It cleaves an **α**1 → 4 glycosidic linkage at the non-reducing end of a starch or malto-oligosaccharide (donor) chain, and transfers the glucose to the non-reducing end of another (acceptor) molecule, predominantly forming **α**1 → 6 glycosidic linkages, yielding mixtures of linear malto-/isomalto-oligosaccharide chains [1, 3, 4]. GTFB also has a relatively low hydrolysis activity. The GTFB final products are very interesting functional carbohydrates, acting as prebiotic oligosaccharides and soluble dietary fiber [5]. The enzymatic conversion of the widely available and low cost substrate starch by GTFB yields potentially valuable food ingredients and provides interesting opportunities for industrial applications [4].

Heterologous expression of GTFB in *E. coli* renders a low amount of soluble GTFB protein and a large amount of GTFB in inclusion bodies (IBs) as described previously for the related GTFML4 enzyme [6]. IBs are generated by protein aggregation, resulting in inactive enzymes, and are commonly observed in heterologous expression systems [7]. Several studies have shown that active enzymes may be isolated from IBs through proper processing [8]. The conventional method involves refolding of the denatured protein. In the first step the misfolded protein is unfolded, followed by a second, gentle, refolding step [9, 10]. Several successful refolding methods have been developed, such as dilution, on-column chromatography, dialysis, ultra-filtration and procedures involving multiple steps [11-13]. Success in protein refolding, however, varies strongly, and these procedures are often time consuming and relatively expensive [14].

Interestingly, in case of GTFB, we detected its enzyme activity in the IBs, which therefore were renamed into non-classical inclusion bodies (ncIBs) [11, 15]. Activity of IB aggregated enzymes may reflect the presence of a percentage of properly folded functional protein [14]. Another viewpoint, the “IB-stretch hypothesis”, poses that IBs may include functional enzyme domains, and that crucial residues for active protein conformation are not engaged in the inactive β-core of the aggregates [16]. These IBs also provide enough pore space for substrate and product molecules [14]. Functional proteins in ncIBs thus can be applied directly; one example is the human granulocyte-colony stimulating factor [15]. These ncIBs may have additional advantages over solubilized protein, such as high stability and relatively large particle size, useful for enzyme immobilization and preparation of nanomaterials [17, 18].

Heterologous expression attempts with *L. reuteri* 4,6-α-glucanotransferase enzymes (e.g. GTFB, GTFW and GTFML4) in *E. coli* yielded low amounts of soluble protein [6]. However, expression of GTFB in *E. coli* BL21 Star (DE3) resulted in an abundant accumulation of GTFB in IBs, as described in this study. Here we report the results of a comparison of soluble GTFB and ncIB GTFB with enzyme preparations obtained by refolding of GTFB protein extracted from IBs, with emphasis on their activity, product specificity and thermostability.

## Results and discussion

### GTFB accumulates as soluble protein and in inclusion bodies (IBs)

Heterologous expression of GTFB in *E. coli* BL21 Star (DE3) resulted in relatively abundant synthesis of this protein but mainly in the form of inclusion bodies. As shown in Table 1, this *E. coli* expression system yielded approximately 150–225 mg of total cell protein per liter of culture, more than 50 % of which was the target GTFB protein. However, more than 90 % of total GTFB protein accumulated in IBs (Table 1).

**Table 1:** The yields of total cell protein, and total, soluble, insoluble GTFB, obtained from *E. coli*

Induction temperature (°C)	Total protein (mg/l culture)	Total GTFB (mg/l culture)	Soluble GTFB (mg/l culture)	Insoluble GTFB (mg/l culture)	% of insoluble GTFB in total IBs
18	168.5 ± 10.0	92.0 ± 5.5	6.0 ± 1.0	86.0 ± 5.0	82.5 ± 0.5
25	175.5 ± 17.5	95.0 ± 9.5	8.0 ± 1.5	87.0 ± 9.0	85.0 ± 0.5
30	224.5 ± 12.5	132.5 ± 7.5	1.5 ± 0.5	131.0 ± 7.0	83.0 ± 0.5
37	150.5 ± 9.0	86.0 ± 5.0	0.5 ± 0.0	85.5 ± 0.5	73.5 ± 0.5

*E.coli* BL21 Star (DE3) cells grown at different temperatures, and the percentages of insoluble GTFB within the inclusion bodies (IBs) were determined by Bio-Rad protein assay and densitometric analysis of SDS-PAGE separated proteins. All cell samples were harvested at a culture OD_600_ of 1.8. The experiments were done in duplicate

Using different induction temperatures, the yields of total protein, soluble and insoluble GTFB protein varied in cultures harvested at the same OD_600_ value of 1.8. The yield of soluble GTFB increased from 0.5 mg/l at 37 °C to 8.0 mg/l at 25 °C, but no further increase occurred at 18 °C. After His-tag affinity purification, only approx. 1 mg pure GTFB was obtained from 1 l culture incubated at 18 or 25 °C. The highest amount of insoluble GTFB (131.0 mg/l) was produced at 30 °C, while the purest (85.0 %) insoluble GTFB was obtained at 25 °C.

### Comparison of hydrolysis and transferase activities of soluble, refolded and ncIB GTFB

**Fig. 1:**
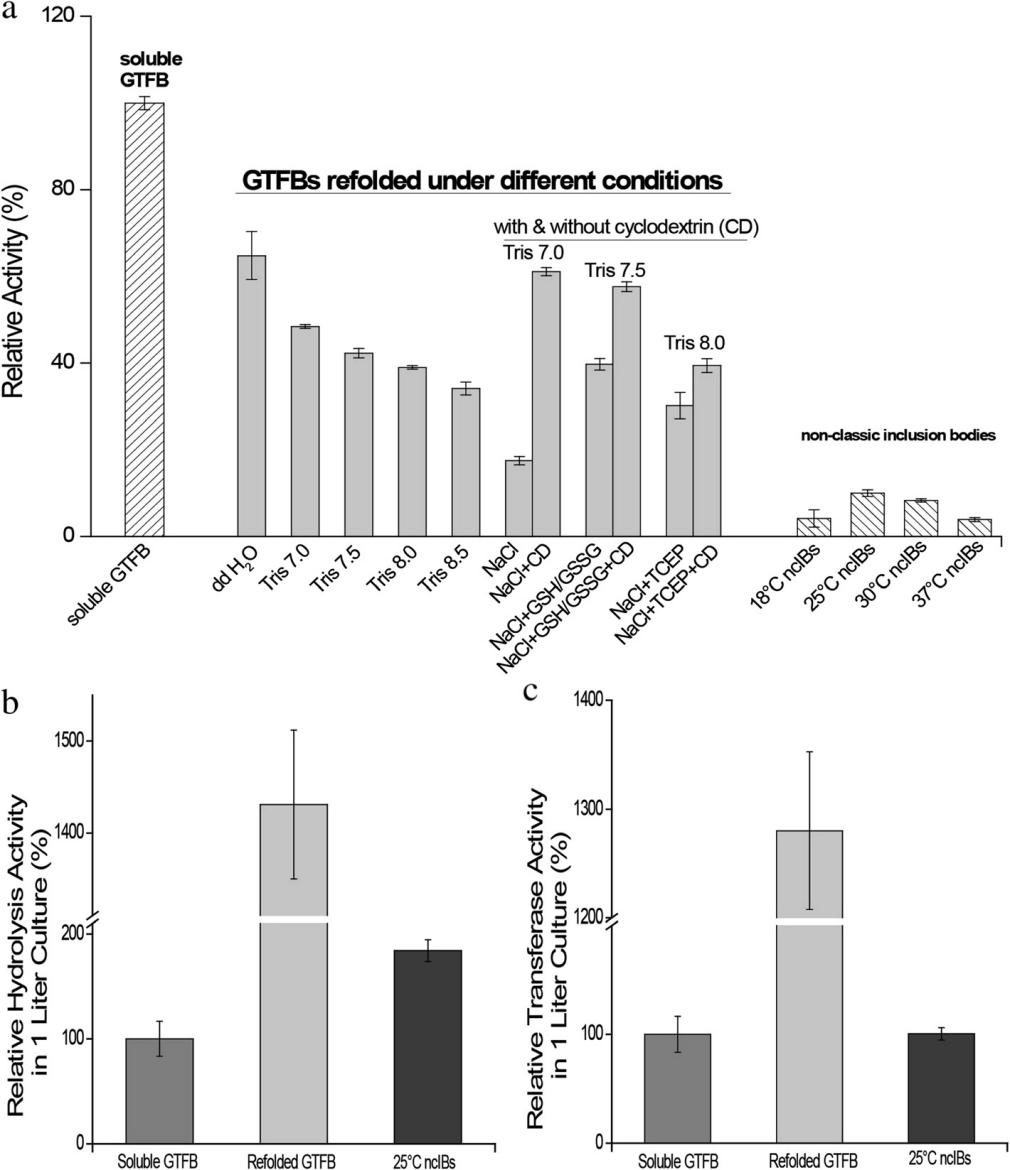
Bargraphs of the relative activities of soluble, refolded and different ncIB GTFB enzymes. The refolded GTFB enzymes were obtained from various refolding buffers, and the non-classical inclusion bodies (ncIBs) GTFB were expressed at different temperatures (**a**). All enzyme concentrations used were 100 μg/ml. The enzyme activity was defined as the release of glucose from maltoheptaose at 37 °C and pH 4.7. The hydrolysis activity of soluble GTFB was set at 100 % (0.17 U). The total hydrolysis and transferase activities of soluble, refolded and ncIB GTFB enzymes obtained from one liter *E. coli* culture expressing GTFB are shown in bargraphs (**b**) and (**c**). In (**b**) 100 % activity corresponds to 13.4 U hydrolysis activity and in (**c**) the 100 % value corresponds to a transferase activity of 4.3 U in 1 l culture

The iFOLD protein refolding matrix with 94 different buffers was used to determine the optimal refolding conditions for GTFB IBs. Refolding efficiency was assessed by measuring hydrolysis activity (glucose release from maltoheptaose). As shown in Fig. 1, refolding was more successful at pH 7.0 and 7.5 compared to pH 8.0 and 8.5. Double distilled (dd) H_2_O at pH 5.0 without any additives and buffer salts was the optimal refolding environment. Through dd H_2_O refolding, 64.8 % hydrolysis activity was recovered compared to soluble GTFB (see Fig. 1). In addition, among all the refolding conditions tested, conditions with methyl-β-cyclodextrin all resulted in higher efficiency than the ones without methyl-β-cyclodextrin (Fig. 1a). Conceivably, the hydrophobic areas of the GTFB protein are exposed during the folding process and addition of methyl-β-cyclodextrin suppresses the stacking of protein folding intermediates [19].

Non-classical inclusion body (ncIB) research shows that the presence of active protein inside IBs strongly depends on the *E. coli* growth temperature [11, 15]. An example is the expression of human granulocyte-colony stimulating factor in *E. coli*: compared to 37 °C expression at 25 °C resulted in higher amounts of correctly folded protein inside IBs [15]. Using equal amounts of protein, four ncIB GTFB samples obtained at different *E. coli* growth temperatures displayed relatively low hydrolysis activity in comparison to the soluble and refolded GTFB enzymes (Fig. 1a). The GTFB activity (hydrolysis) of the most active 25 °C ncIBs GTFB was 10.1 % and 15.6 % of that of the soluble and dd H_2_O refolded GTFB, respectively. To exclude any effect from *E. coli* proteins, *E. coli* harboring the pET15b-gtfBD1015N (encoding inactive GTFB protein) was used as a negative control [1].

Based on the yield of 1 l culture, GTFB ncIBs contained around 180 % hydrolysis activity and 100 % transferase activity compared to soluble GTFB (Fig. 1b and c). Thus ratios of hydrolysis versus transferase activity (Fig. 1b and c) of ncIB and refolded GTFB were relatively high compared to soluble GTFB. The activity obtained from the total yield of refolded protein in one liter culture is much higher (Fig. 1b and c), but the production costs for refolded proteins are estimated to be around 20 times higher than for ncIB proteins [14]. The ncIB GTFB proteins show sufficient activity to replace refolded GTFB protein as functional GTFB enzyme.

### Comparison of product profiles of soluble, refolded and ncIB GTFB enzymes incubated with maltose and maltotriose

**Fig. 2:**
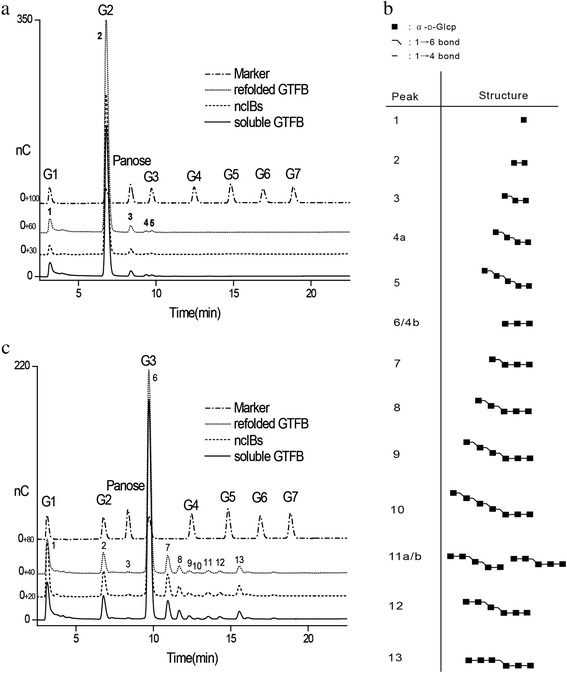
Comparison of HPAEC-PAD profiles of the product mixtures of different GTFB preparations. 50 mM maltose (**a**) and maltotriose (**b**) were seperately incubated with GTFB preparations with equal hydrolysis activity (25.0 μg/ml soluble GTFB, 38.7 μg/ml refolded GTFB and 249.4 μg/ml 25 °C ncIB GTFB) after 72 h at 37 °C and pH 4.7. The oligosaccharide structures produced were identified (**c**) according to Dobruchowska et al. [2]. G1 to G7 represent glucose, maltose, maltotriose, maltotetriose, maltopentaose, maltohexaose and maltoheptaose, respectively

Soluble (25.0 μg/ml), refolded (38.7 μg/ml) and 25 °C ncIB GTFB (249.4 μg/ml) samples with equivalent GTFB hydrolysis activity (0.04 U) were incubated with 50 mM maltose or maltotriose at pH 4.7 and 37 °C for 72 h, and their product profiles were analyzed by HPAEC (Fig. 2a, 2b). The product profiles from soluble, refolded and ncIB GTFB enzymes were very similar. The generated products were identified based on earlier research by Dobruchowska et al. [2]. The structures are depicted in Fig. 2c. In Fig. 2a, two main peaks (1 and 3) identified in all spectra represent glucose and panose, products derived from maltose by hydrolysis and transferase activities. The other reaction products labelled as peak 4a and 5 were panose elongated with one and two (α1 → 6)-linked glucose residues at the non-reducing site. Also after incubation with maltotriose (Fig. 2b), the product profiles of soluble, refolded and ncIB GTFB were highly similar when comparing peaks 7 to 13. The structures of these products (peaks 7–13) indicate that the larger oligosaccharides resulted from elongation with not only α1 → 6 but also α1 → 4 linkages. These data showed that refolded and ncIB GTFB enzymes have the same product and reaction specificity as soluble GTFB, catalyzing hydrolysis and α1 → 4/α1 → 6 transglycosylation.

**Fig. 3:**
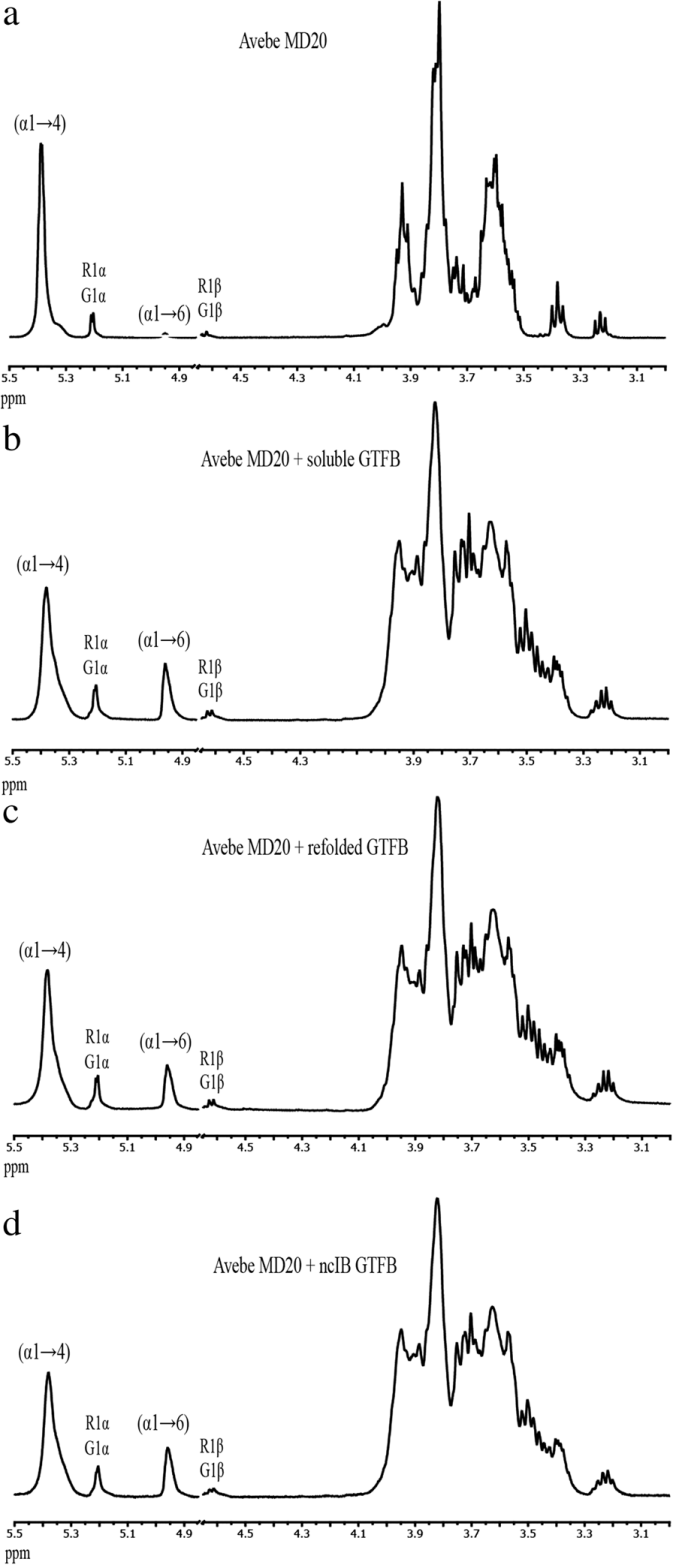
One-dimensional ^1^H NMR spectra of AVEBE MD20 and its products following incubation with GTFB preparations. The spectra were recorded in D_2_O at 300 K after the incubation of 5 % AVEBE MD20 (**a**) with different GTFB preparations which have equal hydrolysis activity (25.0 μg/ml soluble GTFB, **b**, 38.7 μg/ml refolded GTFB, **c** and 249.4 μg/ml 25 °C ncIB GTFB, **d**) after 72 h at 37 °C and pH 4.7. Rα/β represent the reducing –(1 → 4)-D-Glc*p* units and Gα/β represent the D-Glc*p* units

Additionally, NMR analysis (Fig. 3) showed that the α1 → 6 linkage percentages of AVEBE MD20 (maltodextrins manufactured from potato starch, average degree of polymerization 6) modified by soluble, refolded and ncIB GTFB enzymes during incubation for 3 days were highly similar, increasing in all cases from 0.5 to around 15 %.

### Size analysis of AVEBE MD20 and its products after GTFB enzyme treatments

**Fig. 4:**
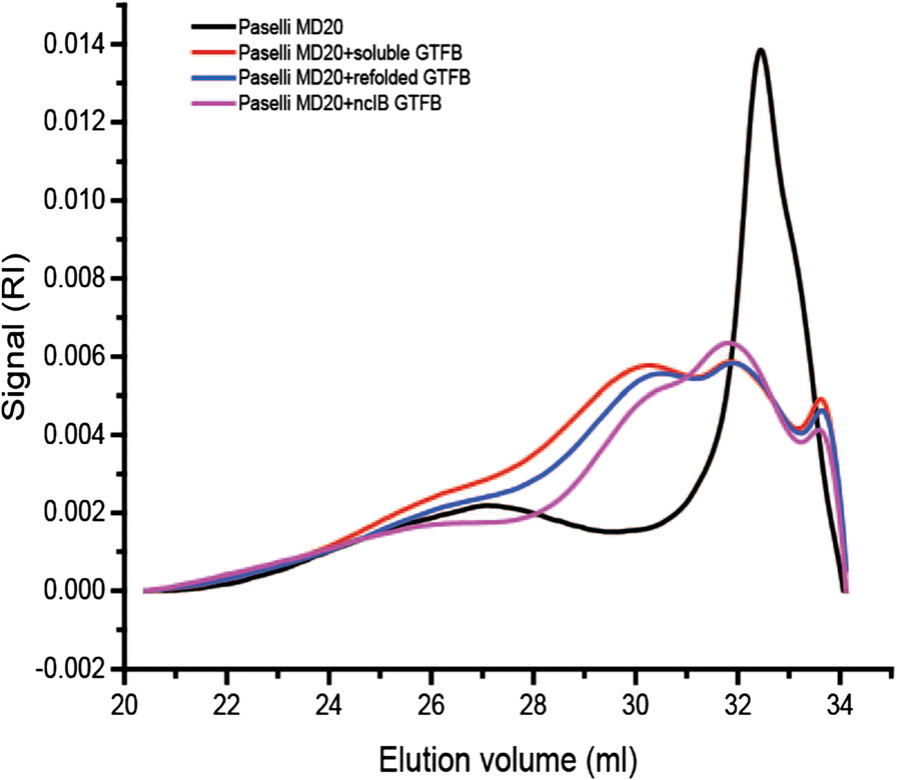
SEC chromatograms of AVEBE MD20 and its products following incubation with GTFB preparations. Different GTFB preparations with equal hydrolysis activity (25.0 μg/ml soluble GTFB, 38.7 μg/ml refolded GTFB and 249.4 μg/ml 25 °C ncIB GTFB) were incubated with 5 % AVEBE MD20 for 72 h at 37 °C and pH 4.7. Elution volumes of the pullulan standards corresponding to 366, 200, 113, 48.8, 21.7, 10, 6.2, 1.32 and 0.342 kDa, are plotted above the x-axis

The Size Exclusion Chromatograms (SEC) of AVEBE MD20 before and after incubation with soluble, refolded and ncIB GTFB proteins are shown in Fig. 4. The elution volume in SEC is directly related to the hydrodynamic volume of the linear and branched molecules [20]. The elution volumes of some pullulan standards are plotted at the x-axis. Since AVEBE MD20 is mainly composed of short oligosaccharides (average degree of polymerization 6), its major peak is seen at a high elution volume (around 32.5 ml). After incubation with GTFB preparations with equivalent hydrolysis activity, the peak at 32.5 ml decreased and shifted to a bi-modal peak at elution volumes of approximately 30 and 32 ml, demonstrating that the short oligosaccharides in MD20 substrate were converted to products with higher molecular weight. The distributions of the products of soluble, ncIB and refolded GTFB were highly similar. The relatively high ratios of hydrolysis versus transferase activity (Fig. 1b and c) of ncIB and refolded GTFB compared to soluble GTFB may initially result in enhanced synthesis of smaller products. This increased availability of shorter acceptor substrates resulted in reduced average sizes of their product molecules.

### Fourier Transform Infrared (FT-IR) studies

**Fig. 5:**
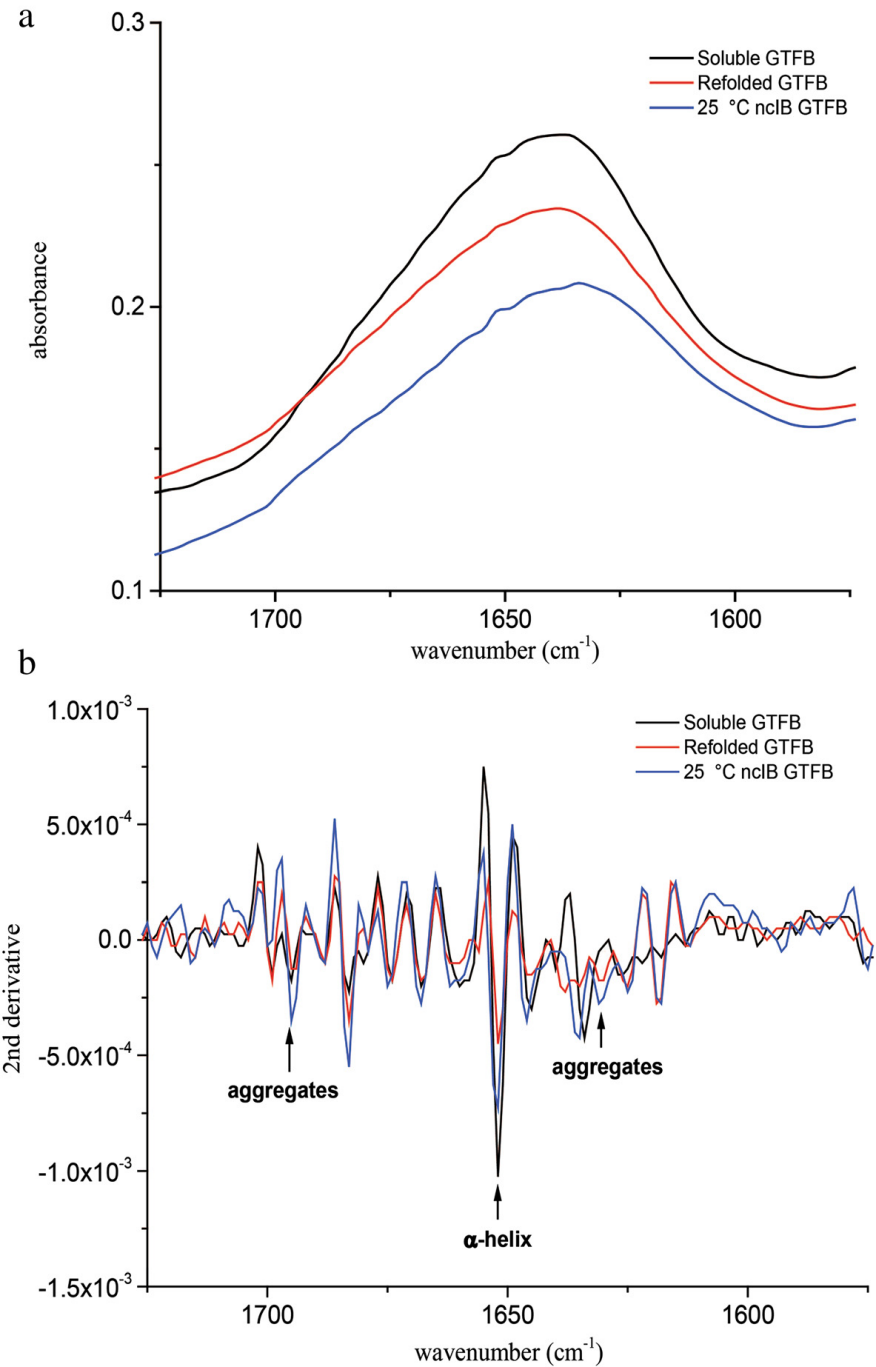
Fourier Transform Infrared (FT-IR) spectra in the amide I region of different GTFB preparations. Soluble GTFB, refolded GTFB, and ncIB GTFB proteins isolated from *E. coli* incubated at different temperatures (25 and 30 °C) were analyzed. (**a**) FT-IR spectra; (**b**) second derivatives of the FT-IR spectra

The secondary structures of the soluble, refolded and ncIB GTFB (25 °C) proteins were examined by infrared spectroscopy (Fig. 5). The frequency and the shape of the amide I bands in the spectral region between 1690 and 1620 cm^−1^ provides information about the type of secondary structure present in proteins [21]. Because the components in amide I, resulting from different secondary structure elements, are strongly overlapping (Fig. 5a), a second derivative analysis was applied. The main band, at 1653 cm^−1^ in second derivative spectra (Fig. 5b) indicates the presence of α-helical structures, which may be expected for a GH70 family enzyme containing a TIM barrel fold with 8 α-helices [22]. The ncIBs GTFB show distinctive bands at 1627 and 1695 cm^−1^ (Fig. 5b) with higher intensity than those of soluble and refolded GTFB, indicating that the inclusion bodies have more intermolecular β-sheet structure [23]. The FI-IR spectra of the inclusion bodies also show peaks at 1633 and 1653 cm^−1^, suggesting they also contain some native β-sheet and native α-helix structures of the soluble GTFB enzyme, respectively [24].

### Thermostability comparison of soluble, ncIB and refolded GTFB proteins

**Fig. 6:**
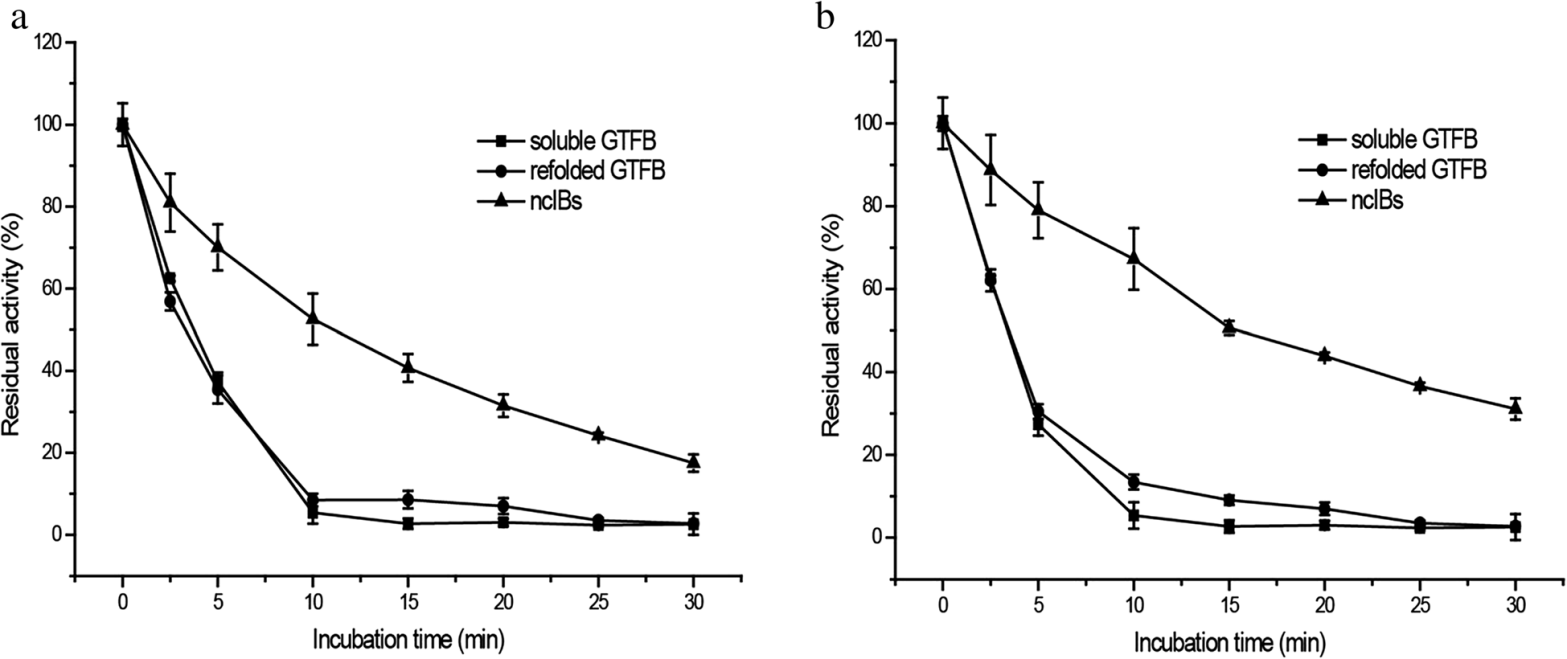
Thermostability of soluble GTFB, refolded GTFB, and 25 °C ncIB GTFB proteins. Different GTFB preparactions incubated at the same protein concentration (75 μg/ml, **a**) and with the same hydrolysis activity (25.0 μg/ml soluble GTFB, 38.7 μg/ml refolded GTFB and 249.4 μg/ml 25 °C ncIB GTFB, **b**), were assessed separately by measuring their residual activities after incubation at 45 °C up to 30 min. The activity before incubation was set at 100 %

To compare the thermostability of soluble, ncIB and refolded GTFB proteins, their residual activity was measured after incubation at 45 °C for several time intervals. At a protein concentration of 75 μg/ml, the half-lives of soluble and refolded GTFB were around 3.5 min (Fig. 6a). The half-life of ncIB GTFB protein was 3.0-fold longer. Even after 30 min incubation, 20 % of the original activity of ncIB GTFB enzyme remained, while soluble and refolded GTFB enzymes had become completely inactivated. When comparing the three GTFB protein preparations in different concentrations but with the same hydrolysis activity (0.04 U), the half-life of ncIB GTFB was 5.0 times longer than those of soluble and refolded GTFB (Fig. 6b). This is probably due to the extended β-sheet formation in ncIB GTFB protein by amino acid chains which are remote to the active site stabilizing its overall conformation. Thus, with increasing temperatures, domains outside the active site important for stable protein conformation may be more slowly affected in ncIB GTFB protein than in soluble and refolded GTFB proteins.

## Conclusions

This paper reports that the *Lactobacillus reuteri* 121 GTFB protein is highly expressed in the heterologous host *E. coli*, but largely accumulates in non-classical inclusion bodies (ncIBs), displaying hydrolysis and transferase activity. *E. coli* growth temperatures of 25 and 30 °C resulted in ncIB preparations with highest GTFB yield, purity and activity. Following denaturing, refolding of GTFB protein in double distilled H_2_O resulted in highest recovery of GTFB (hydrolysis) activity, but required a set of complicated procedures. The ncIB GTFB enzyme produced at 25 °C displayed 10.1 % of the soluble GTFB (hydrolysis) activity and produced similar product profiles from maltose, maltotriose and AVEBE MD20. FT-IR analysis of soluble, refolded and ncIB GTFB proteins confirmed that structural differences exist between these GTFB proteins. The ncIB GTFB enzyme was much more thermostable than the soluble and refolded GTFB enzymes. In view of its high yield, low preparation cost and structural stability, ncIB GTFB protein thus provides a promising option for industrial applications.

## Methods

### Bacterial strains, plasmids, medium and carbohydrate

Plasmid pET15b was used for GTFB [GenBank: AAU08014.2] (C-terminal His-tag) expression in *Escherichia coli* strain BL21 Star (DE3) (Invitrogen, Taastrup, Denmark).

*E. coli* strain BL21 Star (DE3) was grown in Luria-Bertani (LB) medium with 100 mg/l ampicillin and 25 mg/l kanamycin medium. GTFB expression was induced using 0.4 mM Isopropyl β-D-1-thiogalactopyranoside (IPTG). AVEBE MD20 with DE (dextrose equivalent) value 20 was provided by AVEBE (Veendam, The Netherlands). LiBr was purchased from Fisher Scientific and pullulan standards from PSS (Polymer Standard Service, Mainz, Germany). All other materials and chemicals were purchased from Sigma-Aldrich (St. Louis, US).

### Growth and inducing conditions

Soluble GTFB expression in *E. coli* BL21 Star (DE3) was achieved using the procedures described by Kralj et al. [1]. For preparation of IBs, the method was modified as follows: Pregrown *E. coli* BL21 Star (DE3) inoculum was added to 1 l medium in a 5 l shake flask and grown aerobically at 37 °C and 220 rpm until an OD_600_ of 0.9. The culture was equally divided into four 1 l flasks and 0.4 mM IPTG was added to induce GTFB expression. The four flasks of 250 ml broth were incubated at induction temperatures of 18, 25, 30 and 37 °C, respectively. Cultures were harvested at OD_600_ of approx. 1.8. Biomass was collected by centrifugation at 15,000 × g for 30 min.

### Isolation of soluble protein and inclusion bodies

The collected *E. coli* cell pellets from 250 ml culture were washed with buffer (20 mM Tris-Cl, 50 mM NaCl, pH 8.0) and then resuspended in 8 ml of B-PER protein extraction buffer (Thermo, Rockford, US). To lyse *E. coli* cells and to remove nucleic acids, 16 μl lysozyme (150,000 U) and 4 μl DNase (20,000 U) were added and stirred for 30 min at room temperature. The proportions of soluble and total GTFB were determined by densitometric analysis of Coomassie-stained SDS-PAGE gels with 8 μl sample, using a Bio-Rad Model Imaging Densitometer (Bio-Rad Laboratories, Hercules, US). The IBs were washed twice with washing buffer (20 mM Tris-Cl, 0.5 mM EDTA, pH 8.0).

### Purification of soluble GTFB

The extracted soluble GTFB with 6 × His tag was purified by binding to Ni^2+^ nitrilotriacetic acid (Ni-NTA) as described previously [25]. GTFB was eluted with elution buffer (20 mM Tris–HCl, pH 8.0, 200 mM imidazole, 1 mM CaCl_2_), and further purified by anion-exchange chromatography as described previously [1] using a 1-ml HiTrap Q HP column (GE Healthcare, Uppsala, Sweden). Finally, NaCl was removed using a 5-ml Hitrap desalting column (GE Healthcare) run with wash buffer (20 mM Tris–HCl, pH 8.0, 1 mM CaCl_2_) [1].

### iFOLD Protein Refolding system and preparation of refolded GTFB

The iFOLD Protein Refolding system, containing 94 different refolding buffers, purchased from Novagen (Merck KGaA, Darmstadt, Germany), was used to identify the optimal conditions for GTFB refolding.

A total of 0.5 g prepared IBs was dissolved in 12 ml denaturing buffer (20 mM Tris–HCl with 4.47 % N-lauroylsarcosine sodium salt, 5 % glycerol, 50 mM NaCl, 5 mM TCEP, 0.5 mM EDTA, pH 8.0) and stirred till transparent. The solution was centrifuged at 15,000 × g for 15 min. The supernatant was dialyzed twice against 1 l dialysis buffer (10 mM Tris–HCl, 0.06 % N-lauroylsarcosine sodium salt, 0.05 mM EDTA, 0.1 mM TCEP, pH 8.0) at 4 °C for 6 h. At this stage, all GTFB protein was unfolded and no activity could be detected. The protein was refolded by diluting the sample to around 0.1 mg/ml in the different refolding buffers within the iFOLD system, as well as dd H_2_O, followed by stirring overnight at room temperature. Activity of the refolded GTFB protein was assessed by measuring hydrolysis of maltoheptaose, as described below.

Refolded GTFB with 6 × His tag was further purified using Ni-NTA affinity chromatography and anion exchange methods as described previously [1]. Protein purity was assessed on 8 % SDS-PAGE and protein concentrations measured with Bio-Rad protein assay (Bio-Rad Laboratories, Hercules, US) with bovine serum albumin as standard.

### Non-classical inclusion bodies (ncIB) GTFB preparation

The wet IB pellet (0.5 g) was resuspended and homogenized with 10 ml non-denaturing buffer (20 mM Tris–HCl, 0.2 % N-lauroylsarcosine, 20 % glycerol, pH 8.0) by gently pipetting and short sonication for 10 s (Soniprep 150, MSE Ltd, London, UK). The suspension was shaken overnight at room temperature [11]. The ncIB suspensions were stored at 4 °C, and were homogenized every time before use.

To measure protein content, ncIB suspensions were dissolved in denaturing buffer, followed by dialysis against protein storage buffer (20 mM Tris–HCl, pH 8.0) to remove N-lauroylsarcosine which influences the protein dye reagent (Bio-Rad Co. Ltd, US). Concentrations of these dialyzed proteins were measured by the Bio-Rad Protein aDssay. Finally, the concentration of ncIB GTFB was calculated from the total concentration of insoluble protein and the percentage of GTFB (Table 1) which was obtained from the densitometric analysis of Coomassie-stained SDS-PAGE gels.

### Fourier Transformed Infrared analysis

Soluble and refolded GTFB proteins (0.5 - 3 mg) were precipitated from solution by the addition of hydrated ammonium sulfate (final concentration 30 % w/v). ncIBs prepared at various *E. coli* growth temperatures (18, 25, 30, 37 °C), were washed in dd H_2_O. All wet pellets were dried in the Savant DNA Speed-Vac system for 1–2 h prior to analysis to reduce water interference in the infrared spectra. The infrared spectra of protein samples were recorded on a Bruker IFS66S spectrometer equipped with a liquid-nitrogen cooled MTC detector and Golden-Gate ATR diamond cell. The infrared spectra allowed monitoring of the secondary structure of ncIB GTFB protein prepared at different temperatures in comparison with properly folded and soluble GTFB protein. Typically, 64 scans were collected at a resolution of 4 cm^−1^ for all samples. Before the examination of the amide I bands, solvent spectra were recorded and subtracted [11]. The structure of the amide I region was also analyzed by second derivatives.

### Product profiles and enzyme activity assays

The product profiles of the various GTFB preparations were investigated by incubating enzyme in sodium acetate buffer (25 mM, pH 4.7, 1 mM CaCl_2_) at 37 °C for 24 h, with maltose, maltotriose and maltoheptaose separately as substrates [2]. After 24 h or 72 h reactions, all tubes were boiled for 5 min followed by 15,000 × g centrifugation for 5 min. The supernatants were kept for high-pH anion-exchange chromatography (HPAEC) analysis as described below.

GTFB hydrolysis rates were assessed by measuring glucose release. GTFB enzyme solutions (50 μl) with different protein concentrations were added to a final 500 μl reaction system with 10 mM maltoheptaose. At a time interval of 5 min, 50 μl samples were taken and the reaction terminated by addition of 25 μl 0.4 M NaOH, followed by neutralization with 25 μl 0.4 M HCl. The GOPOD kit (Megazyme) was used to detect glucose, as a measure for hydrolysis activity [6]. One unit of hydrolysis activity was defined as the amount of enzyme that produces 1 μmol glucose per min. Transferase activity was measured by determining maltose generation when GTFB enzyme was incubated with amylose (0.25 %, w/v) as donor and glucose (10 mM) as acceptor substrate. One unit of transferase activity was defined as the amount of enzyme that produces 1 μmol maltose per min.

### High-pH anion-exchange chromatography

The reaction products of GTFB were injected onto a 4 × 250 nm CarboPac PA-1 column connected to a Dionex DX500 workstation (Dionex). Samples were run with a gradient of 30–600 mM NaAc in 100 mM NaOH (1 ml/min), and detected by an ED40 pulsed amperometric detector. A mixture with known concentrations of glucose, maltose, panose, maltotriose, maltotetraose, maltopentaose, maltohexaose and maltoheptaose was used as reference.

### Nuclear Magnetic Resonance spectroscopy

NMR spectroscopy resolution-enhanced 1D 500-MHz ^1^H NMR spectra were recorded in D_2_O on a Varian Inova 500 Spectrometer (NMR Center, University of Groningen) at probe temperatures of 300 K. Samples were exchanged twice with D_2_O (99.9 atm% D, Cambridge Isotope Laboratories, Inc.) with intermediate lyophilization and then dissolved in 0.6 ml D_2_O. Chemical shifts (δ) were expressed in parts per million by reference to internal acetone (δ 2.225 for ^1^H).

### Size-exclusion chromatography

DMSO-LiBr (0.05 M) was prepared by stirring for 3 h at room temperature followed by degassing for 15 min an ultrasonic cleaner (Branson 1510, Branson, Danbury, CT). Samples were dissolved at a concentration of 4 mg/ml in DMSO-LiBr by overnight rotation at room temperature, followed by 30 min heating in an oven at 80 °C obtaining clear sample solutions. The samples were cooled to room temperature and filtered through a 0.45-μm Millex PTFE membrane (Millipore Corporation, Billerica, MA). The SEC system set-up (Agilent Technologies 1260 Infinity) from PSS (Mainz, Germany) consisted of an isocratic pump, auto sampler without temperature regulation, an online degasser, an inline 0.2 μm filter, a refractive index detector (G1362A 1260 RID Agilent Technologies), viscometer (ETA-2010 PSS, Mainz) and MALLS (SLD 7000 PSS, Mainz). WinGPC Unity software (PSS, Mainz) was used for data processing. The samples (100 μl) were injected with a flow rate of 0.5 ml/min by an autosampler into a PFG guard column with DMSO-LiBr as eluent. The separation was done by three PFG-SEC columns with porosities of 100, 300 and 4000 Å. The columns were held at 80 °C, the refractive index detector at 45 °C and the viscometer was thermostatted at 60 °C. A standard pullulan kit (PSS, Mainz, Germany) with molecular weights from 342 to 805000 Da was used. The specific RI increment value dn/dc was measured by PSS and is 0.072 (private communication with PSS).

### Thermostability determination

The thermostability of the soluble, ncIB and refolded GTFB proteins with the same concentration (75 μg/ml) and the same hydrolysis activity (0.79 U, 25.0 μg/ml soluble GTFB, 38.7 μg/ml refolded GTFB and 249.4 μg/ml 25 °C ncIB GTFB) were determined by incubation in 25 mM sodium acetate buffer (pH 4.7) at 45 °C. Samples were taken at several time intervals up to 30 min, and the residual hydrolysis activity was determined subsequently as described above.

## Abbreviations

IBs: Inclusion bodies
ncIBs: Non-classical inclusion bodies
GH70: Glycoside hydrolase family 70
SEC: Size exclusion chromatograms
*E. coli*: *Escherichia coli*
